# Nanoengineering of Lu(III) Bisphthalocyanine-Cored Polycaprolactone Polymers and Nanoparticles

**DOI:** 10.1021/acsomega.5c09338

**Published:** 2025-12-19

**Authors:** Atefeh Emami, Heba Z. Alagha, Burak Özdemir, Merve Gülseren, Erdinc Doganci, Ümit İşci, Merve Dandan Doganci, Fabienne Dumoulin

**Affiliations:** † 162328 Acibadem Mehmet Ali Aydinlar University, Graduate School of Natural and Applied Sciences, Ataşehir, 34752 Istanbul, Türkiye; ‡ Acibadem Mehmet Ali Aydinlar University, Faculty of Engineering and Natural Sciences, Department of Biomedical Engineering, Ataşehir, 34752 Istanbul, Türkiye; § 52980 Kocaeli University, Department of Chemistry and Chemical Processing Tech., 41140 Kocaeli, Türkiye; ∥ 52982 Marmara University, Faculty of Technology, Department of Metallurgical & Materials Engineering, 34722 Istanbul, Türkiye

## Abstract

Double-decker (DD) lanthanide complexes of phthalocyanines have peculiar properties, but their structures are rarely functionalized or incorporated into nanomaterials. This work has been conceived to explore the feasibility of using hydroxylated DD complexes as an initiator for Poly­(ε-caprolactone) (PCL) polymerization, with different PCL lengths, and to investigate whether NPs suspended in water can be obtained via the self-encapsulation of these polymers by a nanoprecipitation process. Several structural and experimental parameters have been tested for the nanoprecipitation process to study their potential effects on the NP characteristics.

## Graphical abstract

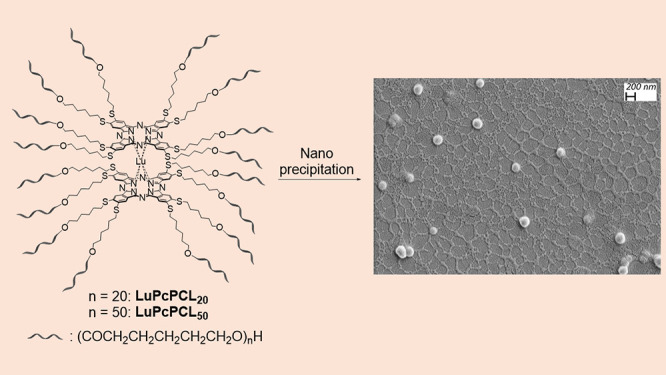

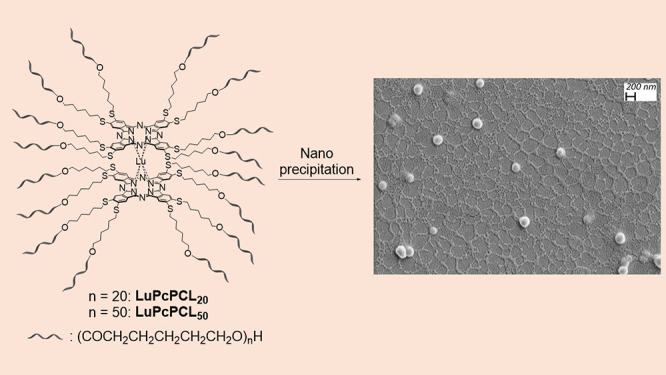

## 1 Introduction

Phthalocyanines (Pcs) are artificial tetrapyrrolic porphyrinoid derivatives that can complex most of the metals and semimetals of the periodic table. In most cases, one Pc macrocycle serves as a single ligand for the complexed cation. Yet, two Pcs are needed to complex a single lanthanide (III) cation, yielding double-decker (DD) complexes, also named sandwich complexes.^1^ Each of these two macrocycles can be identical; then, the complex is said to be homoleptic or can be different, giving so-called heteroleptic complexes. Triple-decker and complexes of higher order can also be obtained.^2^ While the most current applications of Pcs are in catalysis,^3^ photodynamic therapy,^4^ sensors,^5^ and environmental applications such as photovoltaics,^6^ CO_2_ reduction,^7^ hydrogen production,^8^ and methane oxidation,^9^ the peculiar properties of lanthanides DD complexes of Pcs prompt their use in more specific applications.^10^ Their redox properties are indeed especially suitable for SMMs,^11^ redox sensors,^12^ and biosensors^13^ and induce also interesting electrochromic properties.^14^ Their DD architecture, combined with the possibility to obtain specific substitution patterns such as crosswise ABAB motifs, allowed to prepare the first real octupolar cube, which exhibited giant quadratic hyperpolarizability^15^ that could be modulated by complexing different lanthanide ions.^16^ While many efforts focused on obtaining organo-soluble^17^ and water-soluble^18^ derivatives, there are only a handful of functionalized DD lanthanide complexes of Pcs. DD complexes substituted with crown-ethers have demonstrated their interest in various applications such as temperature sensors^19^ and single-molecule magnets (SMMs).^20^ DD with a single pyrene moiety and behaving as supramolecular spin valves^21^ have been efficiently anchored on single-wall carbon nanotubes.^22^ Substitution of Eu, Y and Lu DDs with chiral menthol moieties has produced molecular materials with intense chiral information transfer from the menthol moieties to the Pc macrocycles, which was not observed on the correpsonding monomeric metal-free Pc.^23^ Octahydroxylated^24^ and hexadecahydroxylated^25^ derivatives made of symmetrically substituted Pcs have been synthesized, and we reported the selective preparation of mono and di hydroxylated, mesylated and azido heteroleptic complexes.^26^ To the best of our knowledge, further functionalization of DD is even rarer, which also limits their integration into nanomaterials, despite the obvious interest it would have, especially for biological applications, such as their combination with nanomaterials. Indeed, nanomedicine has bloomed over the last decades, mainly because of the Enhanced Permeation and Retention effect.^27^ Pcs used for biomedical applications—mainly photodynamic therapy—have been incorporated into nanoparticles (NPs) of different nature,^28^ many of them being polymeric.^29^ The biocompatibility of poly­(**ε**-caprolactone) (PCL) polymers has demonstrated their interest for biomedical applications,^30^ and nanoprecipitation is frequently used to encapsulate drugs.^31^ PCL polymers are prepared by using hydroxylated compounds as initiators of ring-opening polymerization (ROP) and fixed amounts of ε-caprolactone (ε-CL), with this amount governing the length of the PCL chains. A handful of hydroxylated Pcs have been used as ROP initiators, producing star polymers.^32^

Optical redox biosensors are important biomedical monitoring and diagnostic tools. Quite naturally, NPs with optical redox biosensing properties have also emerged.^33^ With the aim to obtain nanosize DD for future redox biosensors studies, a complete nanoengineering study aiming at preparing DD-cored star-shaped PCL polymers was conceived to explore the feasibility of using a hexadecahydroxylated DD complex, namely, **LuPcOH**, as an initiator for ε-CL polymerization, with different PCL lengths (Scheme 1), and to investigate whether NPs suspended in water can be obtained via the self-encapsulation of these polymers by a nanoprecipitation process. PCL polymers are known to be used for the encapsulation of several drugs via nanoprecipitation and subsequent drug delivery.^34^ In the present case, the DD complex is encapsulated by the PCL polymers, to which it is covalently attached. Several structural and experimental parameters have been tested for the nanoprecipitation process to study their potential effect on the NP characteristics.

**Scheme 1:**
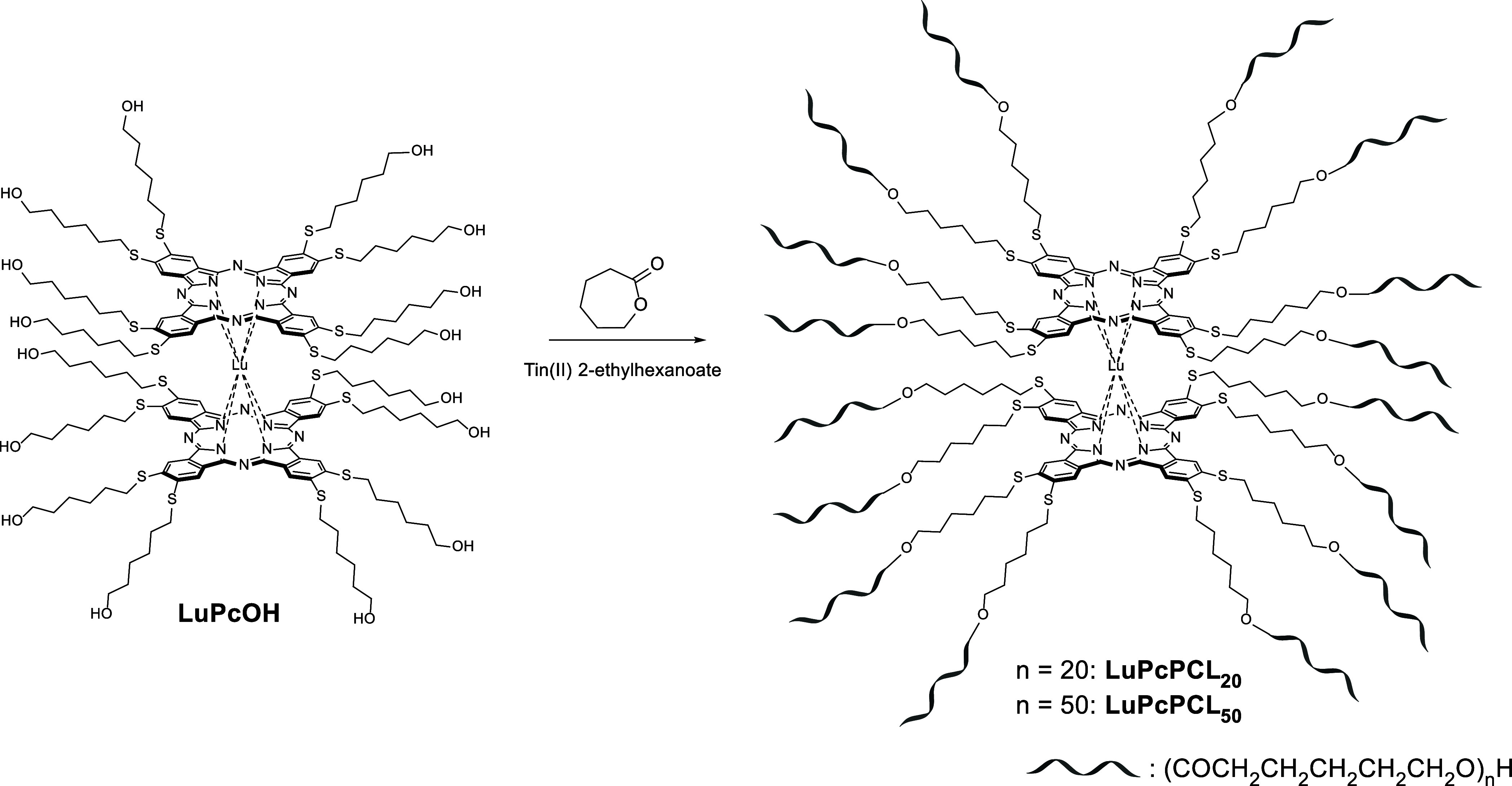
Synthesis of **LuPcPCL**_**20**_ and **LuPcPCL**_**50**_ Using **LuPcOH** as a ROP Initiator

## 2 Experimental Section

### 2.1 Materials

ε-CL with a purity of 97%, obtained from Aldrich, was dried by using calcium hydride (CaH_2_). Prior to use, the ε-CL was distilled and stored under a nitrogen atmosphere. Tin­(II) 2-ethylhexanoate (Sn­(Oct)_2_, 95% purity, Aldrich), dichloromethane (DCM, 99.8% purity, Merck), and methanol (≥99.8% purity, Sigma-Aldrich) were procured from commercial sources and employed without further purification steps. Before use, all solvents and the monomer were purged with nitrogen gas for a minimum of 15 min. 4,5-dichlorophthalonitrile,^35^ **LuPcOH**,^25^ and **LuPcSHex** ^17^ were prepared as previously reported.

### 2.2 Instrumentation and Methods

^1^H NMR spectra were acquired by using a Varian INOVA Unity 500 MHz spectrometer with CDCl_3_ as the solvent. Fourier transform infrared (FT-IR) spectra of all intermediates and copolymers were obtained using an Attenuated total reflectance (ATR) Bruker-Tensor 27 spectrometer in the 4000–600 cm^–1^ range via the attenuated total reflectance (ATR) technique. Gel Permeation Chromatography (GPC) analysis was conducted on a Viscotek Refractive Index (RI) max system equipped with an oven, a pump, an autosampler, and two LT4000L Viscotek T-columns (7 μm particle size, 1500 Å pore size, 300 mm length, and 8 mm inner diameter). Tetrahydrofuran (THF) was used as the eluent at a flow rate of 1.0 mL/min at 23 °C. Polymer concentration was adjusted to 5 mg/mL, and injection volume was adjusted to 100 μL. The calibration curve was constructed with nine polystyrene reference standards with molecular weights ranging from 1200 to 400.000 Da. Data were calculated using the Malvern software program (OmniSEC version 5.12).

The degree of crystallinity (*X*_c_) of synthesized polymers was determined from the DSC data according to the following “eq 1”:

1 $$X_{c}\left(\%\;\mathrm{crystallinity}\right)=\frac{{\Delta H}_{m}}{{\Delta H}_{m}^{0}}\times 100$$

where Δ*H*_m_ is the fusion enthalpy of the polymers. The enthalpy of fusion for perfectly crystalline PCL is given in the literature as Δ*H*_m_^0^ = 139.6 J/g.^36^

Thermal properties were assessed using a Mettler Toledo DSC 1 Star instrument under a nitrogen atmosphere, with heating from room temperature to 220 °C at a rate of 10 °C/min. Thermal decomposition was analyzed using a Mettler Toledo TGA 1 Star System thermogravimetric analyzer from 25 to 650 °C at a heating rate of 10 °C/min under an argon atmosphere.

Average hydrodynamic diameter, polydispersity index (PDI), and average ζ-potential of the nanoparticles in suspension (distilled water) were measured by dynamic light scattering (DLS) using Zetasizer Nano ZS (Malvern Instruments, U.K.) equipped with a 4.0 mV He–Ne laser (633 nm) at 25 °C. The morphology of NPs was examined using scanning electron microscopy (SEM). The samples were visualized with a field-emission scanning electron microscope (Zeiss Leo Supra 35VP, Germany) operated at an accelerating voltage of 3 kV. For each formulation, 10 μL was dropped onto a silicon wafer and dried at room temperature for 2 h. A Nanovak NVTS 400 coater was used to coat the dried samples with gold–palladium (Au–Pd) at 40 mA for 120 s. SEM images were acquired using an In-Lens (IL) detector.

### 2.3 Synthesis of LuPcPCL_20_

**LuPcOH** (26 mg, 0.008 mmol), Sn­(Oct)_2_ (0.005 g, 0.012 mmol), and ε-CL (291 mg, 282 μL, 2.546 mmol) were sequentially placed in a fire-dried reaction flask equipped with a magnetic stirring bar. The reaction mixture was deoxygenated by gentle oxygen-free argon purging for 10 min. The reaction flask was then immersed in an oil bath thermo-stated at 120 °C and stirred for 24 h. The polymerization mixture was then allowed to cool to room temperature. The raw product dissolved in dichloromethane (∼5 mL) was precipitated with cold methanol. **LuPcPCL**_**20**_ was recuperated by filtration through a sintered glass filter (G4) and dried under reduced pressure at ambient temperature until a constant weight was obtained. Yield: 89% (356 mg). FT-IR (cm^–1^): 2942 and 2865 (C–H), 1722 (C=O), 1474 (C–H), 1241 (COO); 1043 (C–O–C). ^1^H NMR (CDCl_3_, δ, ppm): 4.06 (m, -C**H**_2_O­(C=O)−); 3.64 (m, terminal C**H**_2_OH); 2.30 (m, O­(C=O)­C**H**_2_), 1.63 (m, O­(C=O)­CH_2_C**H**_2_CH_2_C**H**_2_CH_2_O­(C=O)), 1.37 (m, O­(C=O)­CH_2_CH_2_C**H**_2_CH_2_CH_2_O­(C=O)).

### 2.4 Synthesis of LuPcPCL_50_

The same procedure as reported above was applied, starting from **LuPcOH** (28.5 mg, 0.009 mmol), and using Sn­(Oct)_2_ (13.9 mg, 0.034 mmol), and ε-CL (784 mg, 762 μL, 6.8 mmol). Yield: 91% (891 mg). FT-IR (cm^–1^): 2946 and 2868 (C–H), 1721 (C=O), 1472 (C–H), 1243 (COO), 1045 (C–O–C). ^1^H NMR (CDCl_3_, δ, ppm): 4.05 (m, -C**H**_2_O­(C=O)−), 3.63 (m, terminal C**H**_2_OH), 2.29 (m, O­(C=O)­C**H**_2_), 1.63 (m, O­(C=O)­CH_2_C**H**_2_CH_2_C**H**_2_CH_2_O­(C=O)), 1.38 (m, O­(C=O)­CH_2_CH_2_C**H**_2_CH_2_CH_2_O­(C=O)).

### 2.5 Preparation of NPs via Nanoprecipitation of **LuPcPCL_m_**

For each polymer, two THF stock solutions (20 and 100 μM) were prepared. The rapid addition of a volume of this as-prepared stock solution (see Table 1) was done to distilled water (2 mL) under vigorous stirring (1200 rpm). The resulting milky mixture was stirred at room temperature for 10 min. No precipitate appears, even after 10 min of stirring. Then, THF was evaporated under vacuum (80 mbar for 15 min at 40 °C). The volume of the final solution was adjusted to 2 mL by adding the necessary amount of distilled water. As the polymers are attached to the DD complex, they are encapsulating, and as the polymers have been purified in a previous step, no surfactant use nor specific purification phase was needed, and the as-prepared solutions were used directly for the next steps.

**Table 1:** Amounts Used for the Preparation of NPs by the Nanoprecipitation of **LuPcPCL**_**m**_

Stock solution (concentration of **LuPcPCL**_**m**_ in THF)	Volume of THF stock solution dropped in water (2 mL)	Final concentration of NPs in water	NPs
20 μM	1 mL	10 μM	**LuPcPCL**_**m**_ **/10**_**from**_ **20**
100 μM	0.2 mL	10 μM	**LuPcPCL**_**m**_ **/10**_**from**_ **100**
100 μM	1 mL	50 μM	**LuPcPCL**_**m**_ **/50**_**from**_ **100**

## 3 Results and Discussion

### 3.1 Synthesis of Polymers

The amount of hexanoate units on PCL polymers is likely to affect their properties as well as those of the NPs going to be prepared hereafter. Two different molar ratios of initiator/ε-CL have been selected, 20 ε-CL/OH and 50 ε-CL/OH. As a result, two different polymers, **LuPcPCL**_**20**_ and **LuPcPCL**_**50**_, have been prepared. The corresponding nonfunctionalized DD with only 16 hexylthio substituents, **LuPcSHex** has also been prepared (see structure in Figure S1) to serve as a reference compound allowing the study of the effect of the presence of the PCL chains on the DD core. **LuPcPCL**_**20**_ and **LuPcPCL**_**50**_ were successfully prepared according to the method in the literature^37^ via ring-opening polymerization (ROP) of ε-CL by using **LuPcOH** as an initiator and tin­(II) octanoate [Sn­(Oct)_2_] as the catalyst, each of the 16 hydroxyls on **LuPcOH** acting as a ROP initiator to yield the star polymers (Scheme 1). No notable difference was observed during both reactions, which proceeded smoothly as in our previous experience,^32b^ and both polymers were obtained in excellent yields in the 90% range.

### 3.2 Structural Characterization of the LuPcPCL_m_ Polymers

Both polymers were first characterized by FT-IR spectroscopy (Figure 1). While **LuPcOH** shows an intense hydroxyl peak, the intensity of the hydroxyl peak of star-shaped PCL polymers after ROP is diminished as the reaction proceeds and as the molecular weight of the polymer increases. Compared to the FT-IR spectrum of **LuPcSHex** that is otherwise very similar, the sharp peak corresponding to the carbonyl group of the ester functions, which appears at 1721 cm^–1^ (C=O) in the spectra of star-shaped PCL polymers, further reflects the incorporation of **ε**-CL monomer in the ROP reaction. These observations confirm that LuPc-cored, star-shaped **LuPcPCL**_**m**_ polymers have been successfully synthesized.

**Figure 1:**
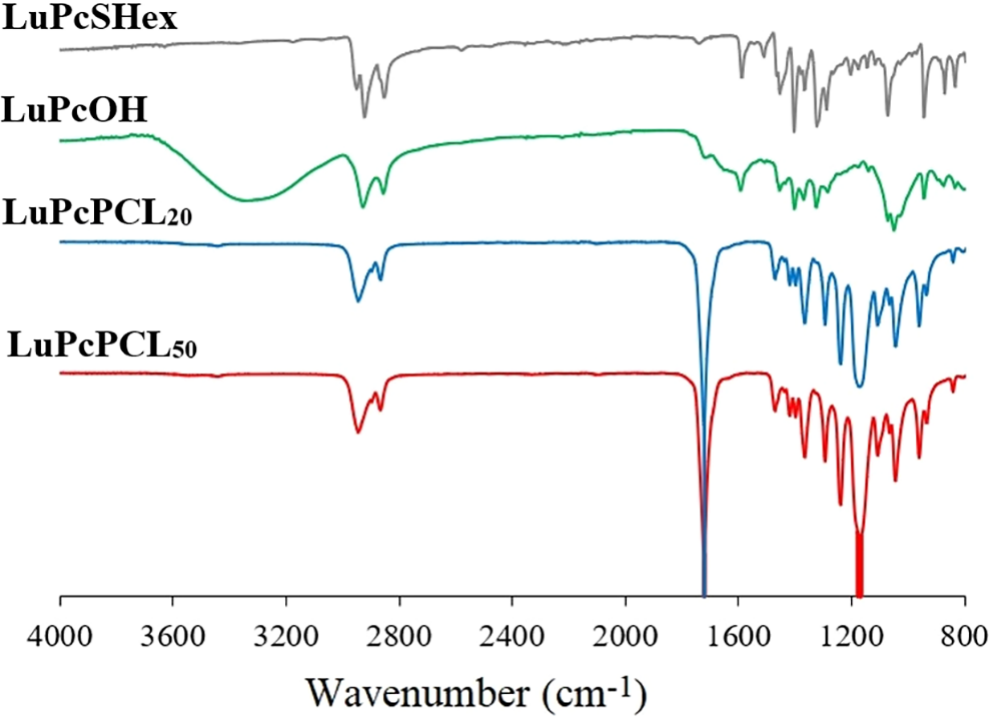
FT-IR of **LuPcSHex** (gray), **LuPcOH** (green), **LuPcPCL**_**20**_ (blue), and **LuPcPCL**_**50**_ (red).

The number-average molecular weights of the obtained **LuPcPCL**_**m**_ polymers as well as the related polydispersity index (PDI) values were determined by GPC (Figure S2 and Table 2). The average molecular weights of **LuPcPCL**_**m**_ increase with the increasing molar ratio of the monomer (ε-CL) to the initiator (**LuPcOH**). Both of the **LuPcPCL**_**m**_ polymers gave symmetrical unimodal elution peaks and had low dispersity, indicating that the purified polymerization products contained only the desired star polymers. ^1^H NMR spectra of star polymers were recorded next in CDCl_3_ (Figure 2). The characteristic methylene protons (H_a_, H_b_, H_c_, and H_d_) of the repeating units in the PCL resonate at 2.30, 1.63, 1.37, and 4.06 ppm, respectively. The degree of polymerization (DP_n_) was found to be 20.72 and 49.51 for each arm of the **LuPcPCL**_**20**_ and **LuPcPCL**_**50**_ polymers, in turn, being equal to the integral ratios between methylene protons of PCL main chain (−CH_2_O­(C=O)−) (H_d_) at 4.06 ppm and methylene groups next to OH groups of PCL (terminal CH_2_OH) (H_e_) at 3.65 ppm (Figure 2). As predicted, the differences in *M*_n,GPC_ and *M*_n,NMR_ values are related to the star-shaped architectures of the produced polymers. Because the hydrodynamic volumes of star-shaped polymers are different than those of linear polystyrene (PS) standards of the same molecular weight, GPC analysis using refractive index (RI) or ultraviolet (UV) detection gives slightly different molecular weights for star-shaped polymers.^38^ As a result, the molecular weights of star-shaped polymers reported by ^1^H NMR are more trustworthy than those established by the GPC analysis.

**Figure 2:**
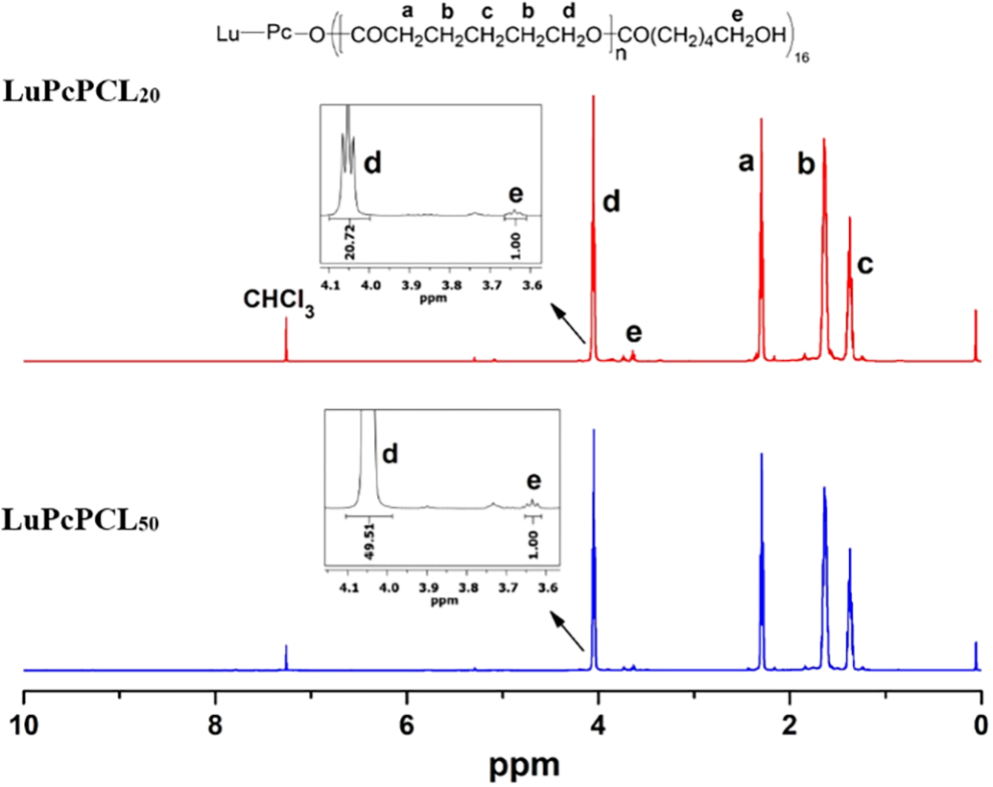
^1^H NMR spectra of **LuPcPCL**_**20**_ and **LuPcPCL**_**50**_ in CDCl_3_.

**Table 2:** Characteristics of the Homo-Armed Star-Shaped **LuPcPCL**_**m**_

	[M]/[I]^a^	*M*_n,NMR_ ^b^ (g/mol)	*M*_n,GPC_ ^c^ (g/mol)	*M*_*w*_ */M*_n_ ^c^
**LuPcPCL**_**20**_	20	41,200	35,300	1.14
**LuPcPCL**_**50**_	50	93,700	98,400	1.23

a M: ε-CL, I: initiator, [M]/[I] = The molar ratio of ε-CL to **LuPcOH** initiator.

b *M*_n,NMR_ was calculated from the integral ratio of the proton signals on the primary hydroxyl methylene end group (-C**H**_2_OH, H_d_) at 3.65 ppm to that of the methylene group on the main chain of PCL (−CH_2_O­(C=O)-, H_e_) at 4.06 ppm from ^1^H NMR analysis.

c *M*_w_/*M*_n_ and *M*_n,GPC_ were determined by GPC analysis. THF was utilized as the eluent with the correction formula: *M*_*n*,*PCL*_ = 0.259 × *M*_n,GPC_^1.073^.^39^

### 3.3 Thermal Properties of the **LuPcPCL_m_** Polymers

Thermal behavior of DD-cored star-shaped PCL polymers **LuPcPCL**_**20**_ and **LuPcPCL**_**50**_ was examined using thermogravimetric analysis (TGA) and differential scanning calorimetry (DSC) and compared to reference **LuPcSHex** and to a linear PCL polymer (**LPCL**). Data are summarized in Table 3 and are shown in Figure 3.

**Figure 3:**
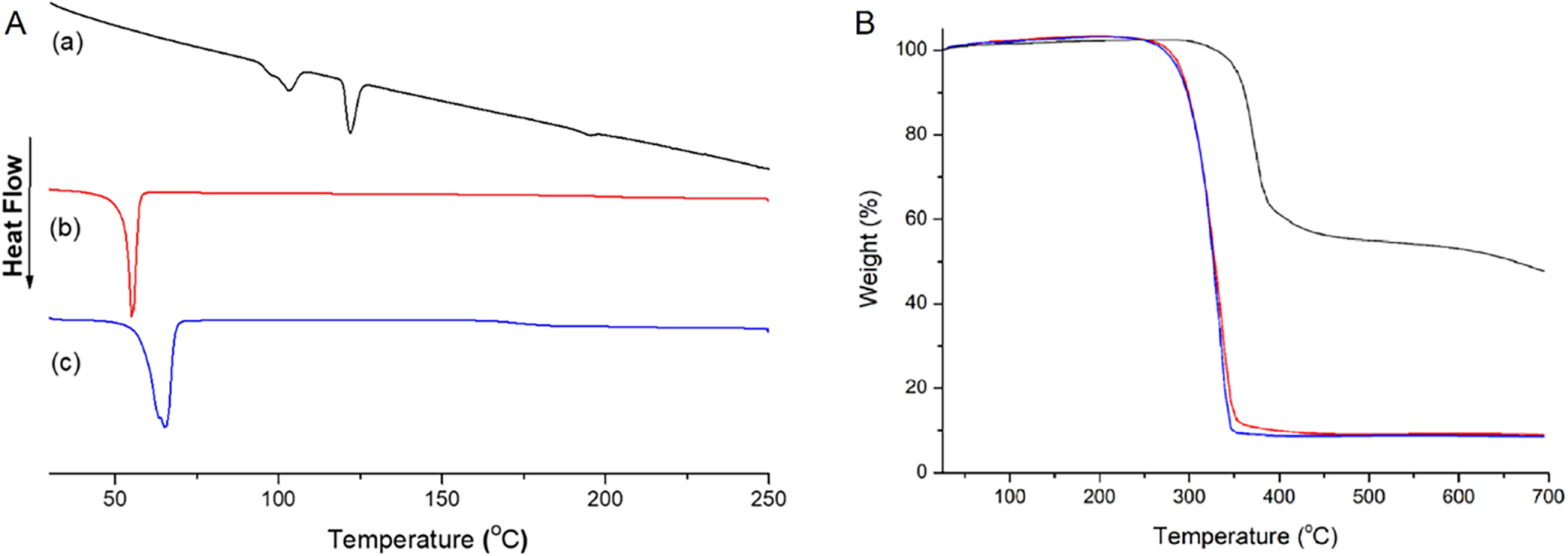
(A) DSC thermograms and (B) TGA thermograms of **LuPcSHex** (black), **LuPcPCL**_**20**_ (red) and **LuPcPCL**_**50**_ (blue).

**Table 3:** Thermogravimetric Analysis Data of **LuPcPCL**_**m**_, and **LPCL** and **LuPcOH** as References

	*T*_m_ (°C)^a^	Δ*H*_m_ (J/g)^b^	*X*_c_ (%)^c^	*T*_onset_ (°C)^d^	*T*_max_ (°C)^e^	Char yield (%)^f^
**LuPcPCL**_**20**_	54.82	99.51	71.28	298	334	9.0
**LuPcPCL**_**50**_	65.04	92.70	66.40	297	333	8.7
**LuPcOH**	N/A	N/A	N/A	363	374	47.8
**LPCL** ^38a^	57.19	122.52	87.76	313	352	4.5

a *T*_m_: maximal melting temperature in the first heating run.

b Δ*H*_m_: fusion enthalpy of the polymers in the first heating run.

c *X*_c_ = Δ*H*_m_ /Δ*H*_m_^0^: enthalpy of fusion for perfectly crystalline PCL. Δ*H*_m_^0^ = 139.6 J/g.^36^

d *T*_onset_: onset decomposition temperature.

e *T*_max_: temperature corresponding to the maximum rate of weight loss.

f Percent of char yield at 700 °C.

First, the melting and crystallization characteristics of all species were investigated by using DSC. The typical DSC curves of the **LuPcPCL**_**m**_ star-shaped polymers and of **LuPcSHex** under the first heating run are shown in Figure 3A. Table 3 provides data related to peak melting points (*T*_m_), fusion enthalpies (Δ*H*_m_), and crystallinity (*X*_c_) of these polymers, as well as the related data of the **LPCL** to provide a comparison for characteristic melting and crystallization transitions typical of semicrystalline linear PCL polymers.

**LPCL** and all star-shaped polymers have a monomodal melting peak at *T*_m1_ = 57.19 to 65.04 °C in the first heating run. The maximum melting point (*T*_m_) increases with the increase in polymer molecular weight, while the Δ*H*_m_ and *T*_c_ values decrease. For star-shaped PCLs, increasing molecular weight elevates the maximum melting point (*T*_m_) due to enhanced crystalline perfection and thermodynamic stability resulting from longer chain segments, concurrently reducing the overall crystallinity (Δ*H*_m_) as a consequence of increased chain entanglement and conformational restrictions. Moreover, *X*_c_ and Δ*H*_m_ values of **LPCL** were higher than those of the obtained star-shaped polymers. The highest degree of crystallinity was observed for **LPCL** with a 87.76% value, as the linear chains of **LPCL** can easily fold and pack regularly into a crystalline lattice due to their high degree of segmental freedom and simple structure. *X*_c_ values were determined as 71.28 and 66.40% for **LuPcPCL**_**20**_ and **LuPcPCL**_**50**_, respectively. In star-shaped polymers, multiple PCL arms radiate from a central core. The core and the high concentration of chain ends restrict the molecular mobility of the polymer segments, making it more difficult for the chains to align, fold, and pack efficiently into ordered crystallites. This leads to a reduction in the overall degree of crystallinity and often results in smaller or less regular crystallites. Crystal defects can also result from an increased number of hydroxyl groups and covalent bonding of PCL arms to the LuPc core. Because the arms of the star-shaped polymers were attached to a LuPc core, chain motions were restricted and the crystallinity of the star polymers decreased.

The thermal stability of **LuPcPCL**_**20**_ and **LuPcPCL**_**50**_ was evaluated via TGA analysis. The curves of percent weight loss against temperature are plotted in Figure 3B, whereas data related to *T*_onset_, *T*_max_, and percent char yield are summarized in Table 3. It appears that the presence of the PCL arms decreases the overall thermal stability of these star polymers compared to reference **LuPcSHex**, without being related to the PCL arm lengths on **LuPcPCL**_**20**_ and **LuPcPCL**_**50**_, which both exhibit the same thermal behavior. It is widely assumed that the thermal degradation of PCL polymers is caused by the presence of the thermally unstable terminal hydroxyl groups.^40^ Compared to **LPCL** initiated by ethanol, **LuPcPCL**_**m**_ has a higher char yield owing to the inherent stable behaviors of the LuPc core itself.^32a,37a^ While the addition of the LuPc core significantly enhanced the char yield of the star-shaped polymers, the *T*_onset_ and *T*_max_ of these polymers were reduced due to the increased number of PCL arms relative to LPCL, specifically the number of terminal hydroxyl end group.

### 3.4 UV–Vis Properties of the **LuPcPCL_m_** Polymers

Ultraviolet–visible (UV–vis) properties of phthalocyanines are among the chief reasons for their interest in many applications. It is crucial to comment on their solubility and possible aggregation state and to follow changes induced during their use related to their applications. Both polymers are soluble in organic solvents such as chloroform and THF, in which the reference compound **LuPcSHex** is also soluble. UV–vis spectra of the three were recorded in 2–10 μM (Figure 4). While the length of the PCL chains does not affect the UV–vis absorption properties of both polymers, the presence of the PCL chains affects in the same extent these properties, with a notable decrease in their extinction coefficient value (the maximum of the Q-band remaining centered at 700 nm for all). One can assume that the presence of the PCL chains has a hypochromic effect, as previously observed.^41^

**Figure 4:**
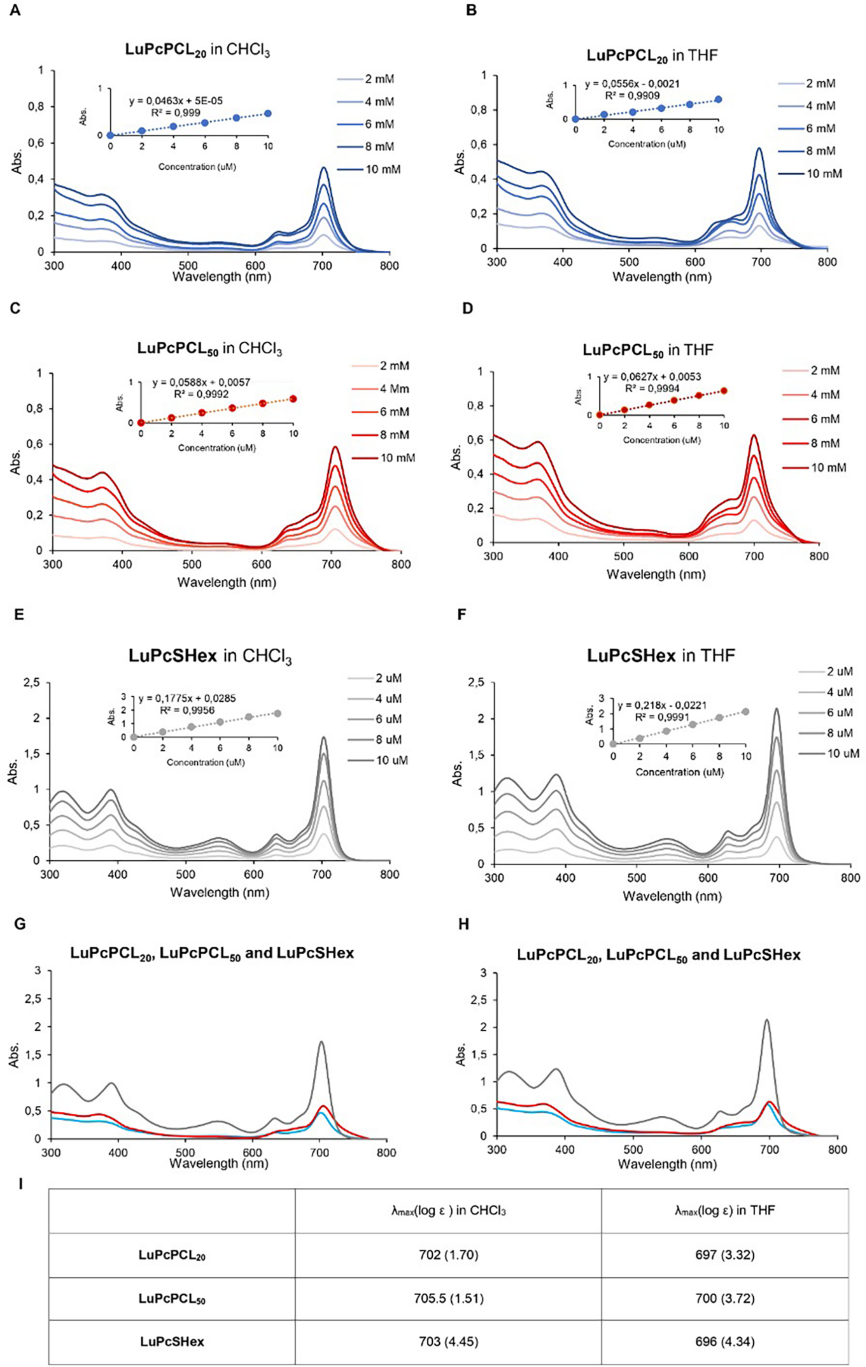
UV–vis spectra of **LuPcPCL**_**20**_ (A, B), **LuPcPCL**_**50**_ (C, D), and **LuPcSHex** (E, F) in the 2–10 μM concentration range. Superimposed spectra show maximum absorption wavelength (Q-band) and related extinction coefficient (I).

### 3.5 Preparation of NPs via Nanoprecipitation

Many factors can affect the characteristics of NPs prepared by nanoprecipitation,^42^ including for PCL-based nanomaterials.^43^

Three different experimental conditions have been defined and used for the preparation of NPs from each polymer; hence, 6 types of NPs were prepared in total. As the **LuPcPCL**_**m**_ polymers are well soluble in THF, it was selected as the preferred water-miscible solvent. To check the effect of the concentration of the polymers in the initial THF stock solutions, two stock solutions of polymers in THF have been prepared, either 20 or 100 μM. From those:

- Two solutions of NPs with final 10 μM Pc concentrations have been prepared, from each of these stock solutions, yielding four NPS: **LuPcPCL**_**20**_ **/10**_**from**_ **20**, **LuPcPCL**_**50**_ **/10**_**from**_ **20**, **LuPcPCL**_**20**_ **/10**_**from**_ **100**, and **LuPcPCL**_**50**_ **/10**_**from**_ **100**
- One solution of NPs (**LuPcPCL**_**20**_ **/50**_**from**_ **100** and **LuPcPCL**_**50**_ **/50**_**from**_ **100**), with the final 50 μM Pc concentration prepared from the 100 μM stock solution.

Similar experimental protocol was followed for the preparation of all NPs, as previously used.^32a,44^ The addition of a suitable volume of the NPs in THF was rapidly carried out under vigorous stirring, the THF was then evaporated under mild conditions, and the volume of the resulting suspensions was adjusted to 2 mL. No precipitation was observed during the whole procedure, and no difference was observed in the behavior of the NPs relative to the polymer used for their preparation.

### 3.6 Size, Stability, and Morphology of the NPs

The hydrodynamic diameter of all the NPs was measured right after their preparation (Figure 5, Tables 4 and S1–S7). Only one population of NPs was observed for each sample, with a low polydispersity index (PDI) for all NPs, showing their homogeneity.

**Figure 5:**
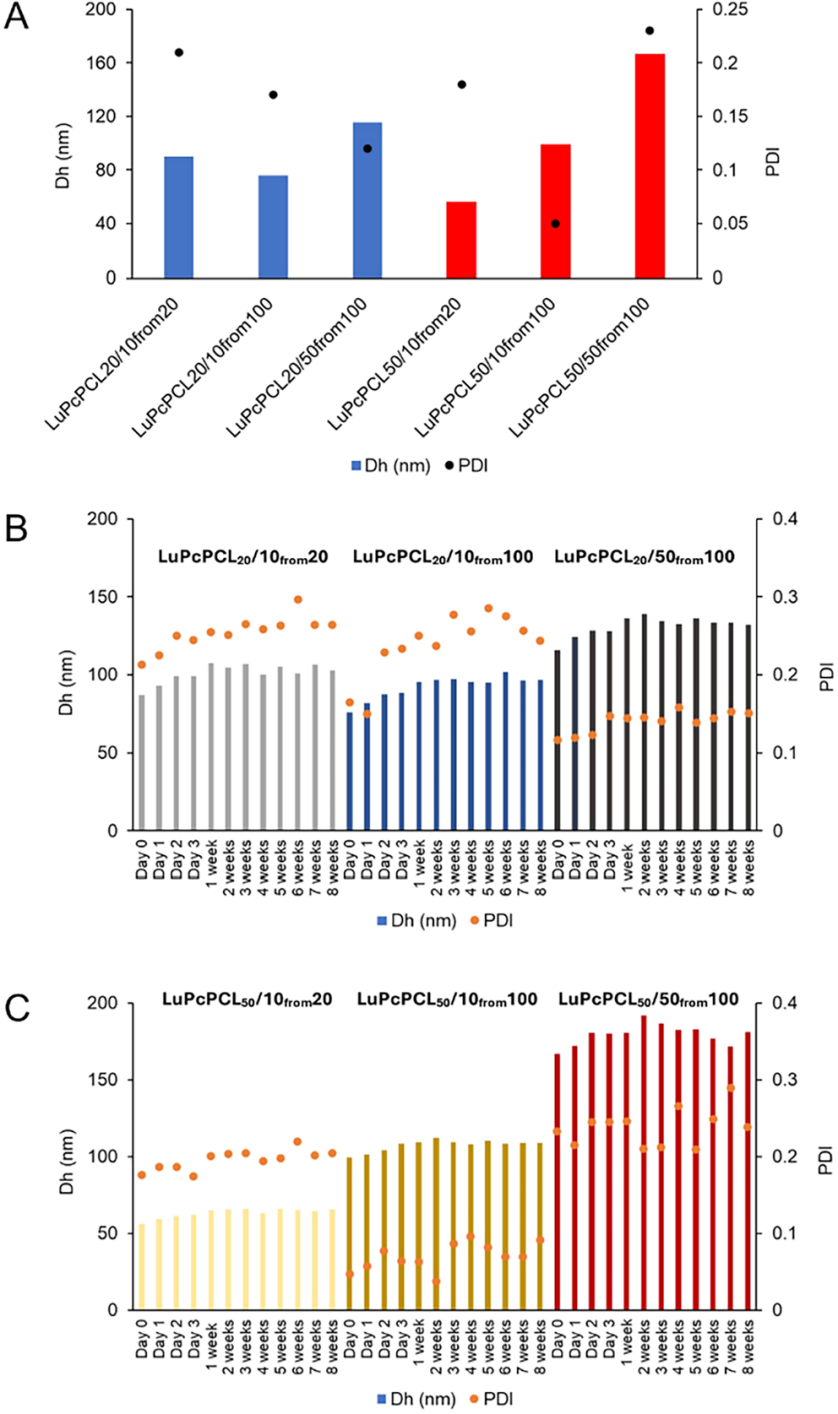
Initial size and PDI of NPs measured by DLS (A), size and PDI of **LuPcPCL**_**20**_ (B) and of **LuPcPCL**_**50**_ (C) measured over 8 weeks.

**Table 4:** Size, Polydispersity, and ζ-Potential Values for the Nanoparticles

	Size (nm)	PDI	ζ-potential (mV)
**LuPcPCL**_**20**_ **/10**_**from**_ **20**	87	0.21	–38.1
**LuPcPCL**_**20**_ **/10**_**from**_ **100**	76	0.17	–42.9
**LuPcPCL**_**20**_ **/50**_**from**_ **100**	116	0.12	–27.5
**LuPcPCL**_**50**_ **/10**_**from**_ **20**	56	0.18	–30.8
**LuPcPCL**_**50**_ **/10**_**from**_ **100**	99	0.05	–29.2
**LuPcPCL**50/50_**from**_ **100**	167	0.23	–51.5

The way the NPs were prepared appeared to have more effect on their size than the number of CP units, the final concentration being the critical parameter rather than the initial concentration in the stock solution. For both final concentrations of 10 μM, prepared from either a 20 μM or a 100 μM stock solution, the size of the NPs is nearly the same, while more concentrated NPs have a larger size. One may hypothesize that more concentrated NPs have more chance to interact and/or that their chains are more likely to deploy and cosolubilize each other. Next, the stability of the NPs was monitored over 8 weeks by DLS, by recording their size and PDI. Only small variations could be observed, showing the excellent stability of these suspensions (Figure 5B,C and Tables S1–S7).

The stability of the nanoparticles, already confirmed by DLS measurements over 8 weeks, was further investigated by measuring their ζ-potential. All nanoparticles are significantly negatively charged in aqueous media, with ζ-potential values ranging from −27 to −51 mV (Table 4). Such values indicate a colloidal suspension stability^45^ by creating strong electrostatic repulsion between particles, in line with the DLS observations and previous reports of PCL-based nanoparticles prepared by nanoprecipitation.^46^

Finally, the morphology of the NPs was examined by SEM microscopy, showing the round shape of the NPs (Figure 6), as well as their homogeneity in size, which is crucial for future applications needing homogeneous samples.

**Figure 6:**
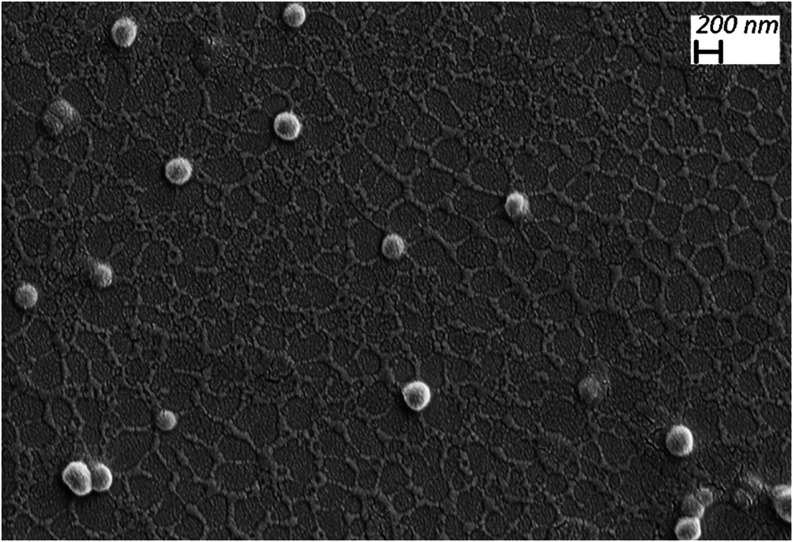
SEM microphotograph of the **LuPcPCL**_**20**_ **/50**_**from**_ **100**.

## 4 Conclusions

A multihydroxylated specific type of Pc complex with peculiar properties, a lanthanide double-decker complex with 16 substituents each ending with a hydroxyl functional group, was selected to be used as a multiple ROP initiator for the polymerization of ε-caprolactone. It’s successfully yielded LuPc-cored star polymers with 16 PCL arms with different PCL content. The full characterization of these organo-soluble polymers showed that the spectroscopic properties of the LuPc core are retained with a slight hypochromic effect. These star polymers next underwent a nanoprecipitation process to yield biocompatible LuPc-based NPs colloidal suspensions in water. Different nanoprecipitation conditions were tested. All resulting NPs exhibited excellent homogeneity in size and morphology, as well as a long stability over time, reflected by the ζ-potential values and DLS measurements over 8 weeks. While obtaining functionalized DD Pc complexes is challenging and such materials remain rare, this work is a proof of concept opening new opportunities toward the use of these nanomaterials for future applications.

## Supplementary Material



## Notes

Structure of the reference compound **LuPcSHex** GPC elugrams of **LuPcPCLm** polymers. DLS spectra and ζ-potential distribution spectra for all NPs (PDF)
