# Supplementary material for: Nanoengineering of Lu(III) Bisphthalocyanine-Cored Polycaprolactone Polymers and Nanoparticles
Source: ACS Omega. 2025 Dec 19;11(1):1600–9. doi: 10.1021/acsomega.5c09338 (PMC12809349; doi:10.1021/acsomega.5c09338)
Supplement: Supplementary file 1 [file ao5c09338_si_001.pdf]

Nanoengineering of Lu(III) Bisphthalocyanine-Cored Polycaprolactone Polymers and Nanoparticles

Atefeh Emami,[1] Heba Alagha,[2] Burak Özdemir,[2] Merve Gülseren,[2] Erdinc Doganci,[3] Ümit İşci,[4] Merve Dandan Doganci[*3] and Fabienne Dumoulin[*1,2]

1 Acibadem Mehmet Ali Aydinlar University, Graduate School of Natural and Applied Sciences, Ataşehir, Istanbul, Ataşehir, 34752 Istanbul, Türkiye

2 Acibadem Mehmet Ali Aydinlar University, Faculty of Engineering and Natural Sciences, Department of Biomedical Engineering, Ataşehir, 34752 Istanbul, Türkiye

3 Kocaeli University, Department of Chemistry and Chemical Processing Tech.,

41140 Kocaeli, Türkiye

4 Marmara University, Faculty of Technology, Department of Metallurgical & Materials Engineering, 34722 Istanbul, Türkiye

Content

**Figure S1.** Structure of the reference compound **LuPcSHex**	pp 2
**Figure S2**. GPC elugrams of **LuPcPCLm** polymers.	pp 2
**Table S1**. Diameter hydrodynamic (Dh) determined by DLS and PDI for all NPs	pp 3
**Tables S2-S7**. DLS spectra and size distributions by number recorded over 8 weeks for all NPs	pp 3-8
**Table S8.** Zeta potential distribution spectra of the nanoparticles	pp 9

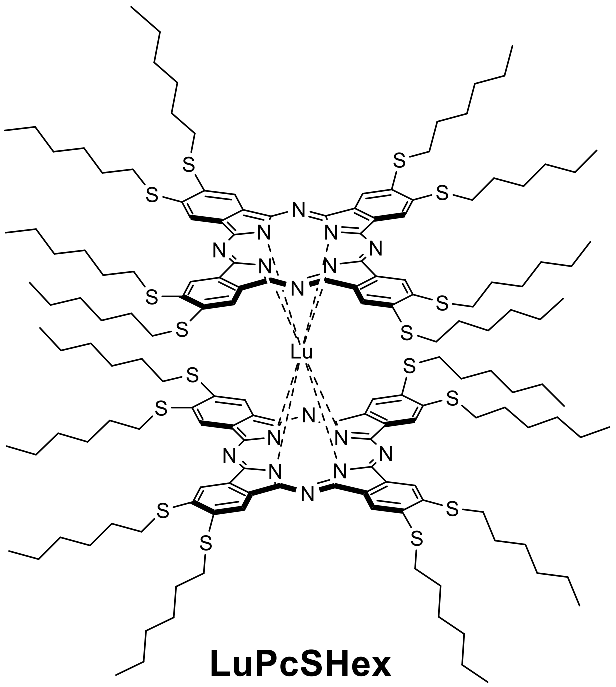

Figure S1. Structure of the reference compound **LuPcSHex**

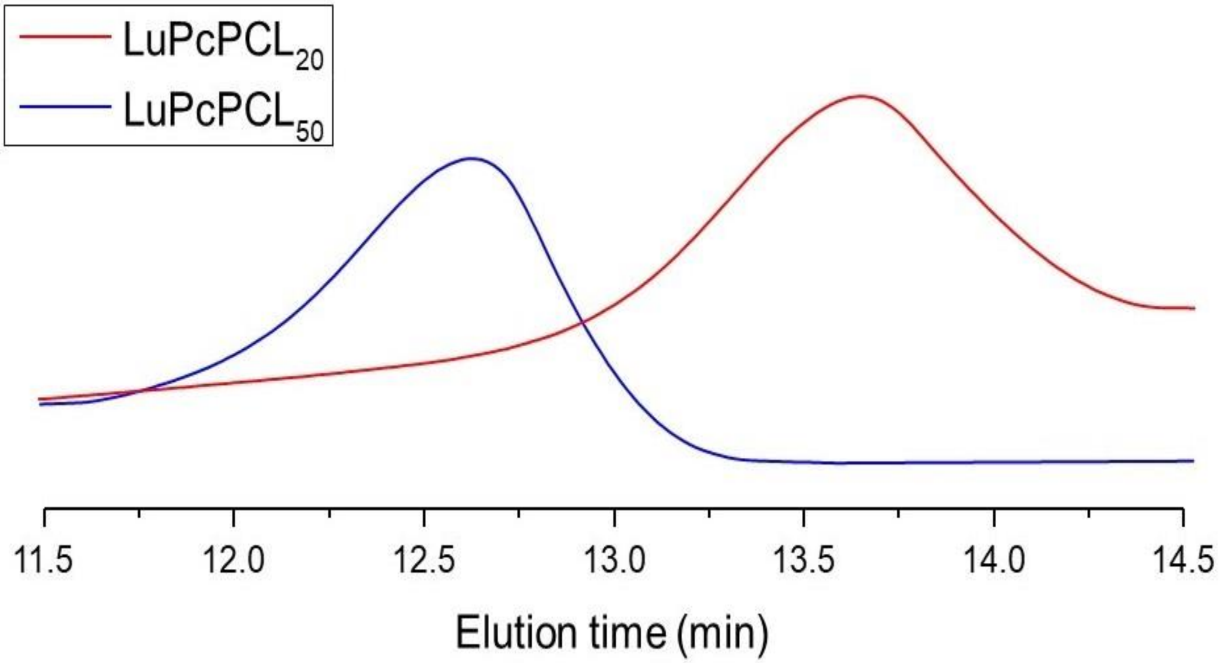

Figure S2. GPC elugrams of **LuPcPCLm** polymers.

Table S1. Diameter hydrodynamic (Dh) determined by DLS and PDI for all NPs

	Dh (nm) / PDI											
	0 h	24 h	48h	72 h	1 week	2 weeks	3 weeks	4 weeks	5 weeks	6 weeks	7 weeks	8 weeks
**LuPcPCL$_{20}$/10$_{from}$20**	86.95 / 0.21	93.14 / 0.23	99.19 / 0.25	98.90 / 0.25	107.63 / 0.26	104.67 / 0.25	106.90 / 0.27	99.91 / 0.26	104.97 / 0.26	100.17 / 0.30	106.40 / 0.26	103.03 / 0.26
**LuPcPCL$_{20}$/10$_{from}$100**	75.70 / 0.17	81.75 / 0.15	87.63 / 0.23	88.55 / 0.23	95.23 / 0.25	96.94 / 0.24	97.36 / 0.28	95.40 / 0.26	94.92 / 0.29	101.82 / 0.28	96.14 / 0.26	96.88 / 0.24
**LuPcPCL$_{20}$/50$_{from}$100**	115.80 / 0.12	124.40 / 0.12	128.17 / 0.12	127.90 / 0.15	136.47 / 0.14	139.13 / 0.15	134.53 / 0.14	132.67 / 0.16	136.17 / 0.14	133.60 / 0.14	133.33 / 0.15	131.97 / 0.15
**LuPcPCL$_{50}$/10$_{from}$20**	56.02 / 0.18	59.36 / 0.19	61.10 / 0.19	62.21 / 0.17	64.75 / 0.20	65.61 / 0.20	65.93 / 0.20	62.90 / 0.19	65.07 / 0.20	65.06 / 0.22	64.53 / 0.20	65.35 / 0.20
**LuPcPCL$_{50}$/10$_{from}$100**	99.34 / 0.05	101.17 / 0.06	104.03 / 0.08	108.20 / 0.06	109.10 / 0.06	111.90 / 0.04	109.33 / 0.09	107.90 / 0.10	110.43 / 0.08	108.43 / 0.07	108.57 / 0.07	108.60 / 0.09
**LuPcPCL$_{50}$/50$_{from}$100**	166.80 / 0.23	172.17 / 0.22	180.46 / 0.25	180.13 / 0.25	180.57 / 0.25	192.03 / 0.21	186.53 / 0.21	182.57 / 0.27	182.67 / 0.21	176.9 / 0.25	171.70 / 0.29	186.17 / 0.24

Table S2. DLS spectra and size distributions by number recorded over 8 weeks for **LuPcPCL$_{20}$/10$_{from}$20**

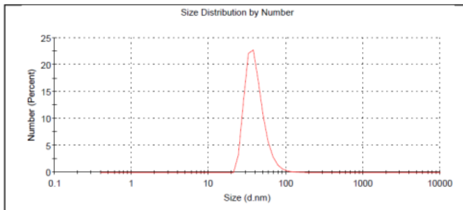

Just after preparation / day 0

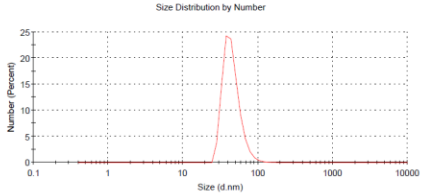

24 h / day 1

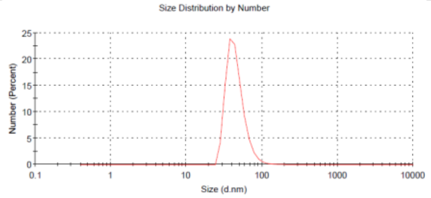

48 h / day 2

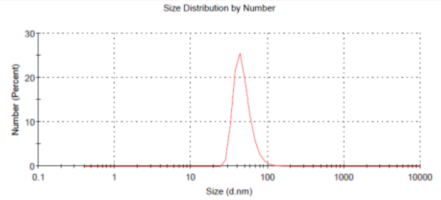

72 h / day 3

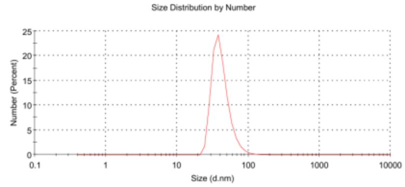

After one week

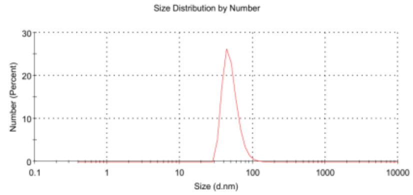

After two weeks

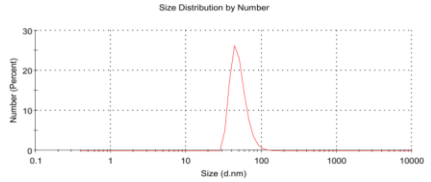

After three weeks

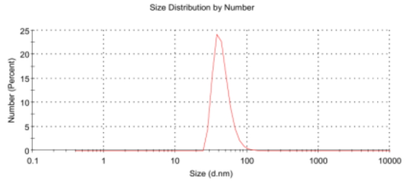

After four weeks

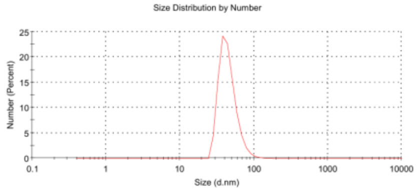

After five weeks

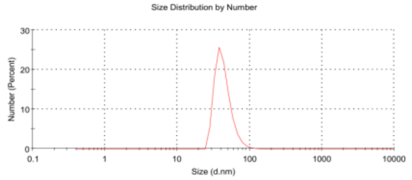

After six weeks

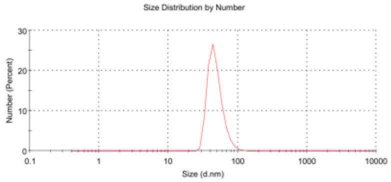

After seven weeks

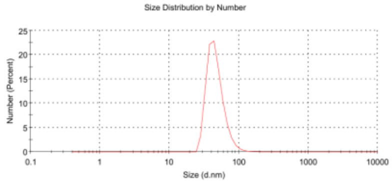

After eight weeks

Table S3. DLS spectra and size distributions by number recorded over 8 weeks for **LuPcPCL$_{20}$/10$_{from}$100**

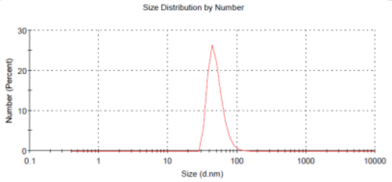

Just after preparation / day 0

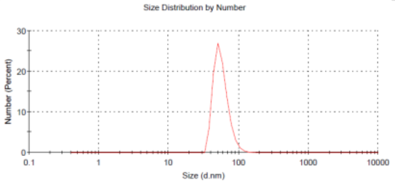

24 h / day 1

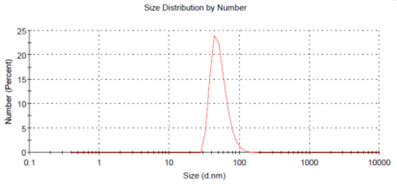

48 h / day 2

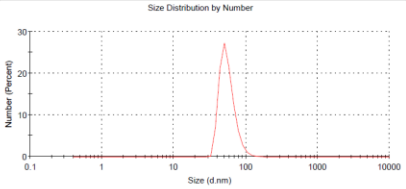

72 h / day 3

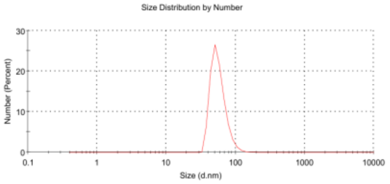

After one week

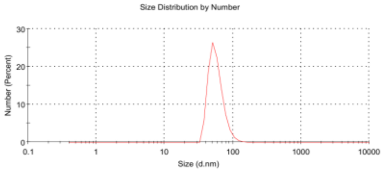

After two weeks

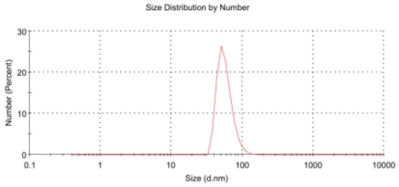

After three weeks

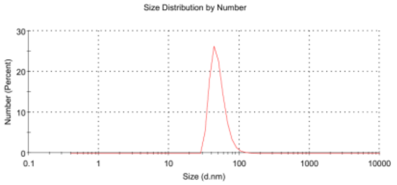

After four weeks

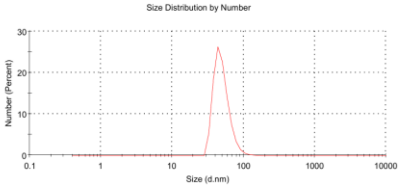

After five weeks

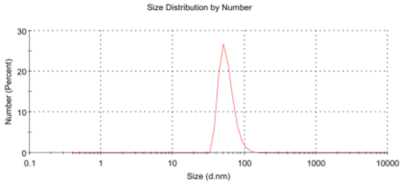

After six weeks

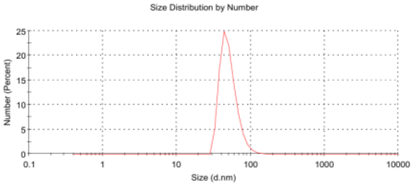

After seven weeks

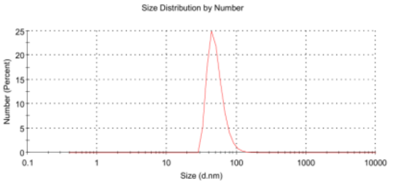

After eight weeks

Table S4. DLS spectra and size distributions by number recorded over 8 weeks for **LuPcPCL$_{20}$/50$_{from}$100**

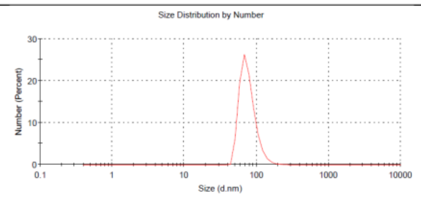

Just after preparation / day 0

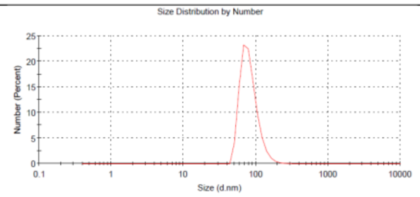

24 h / day 1

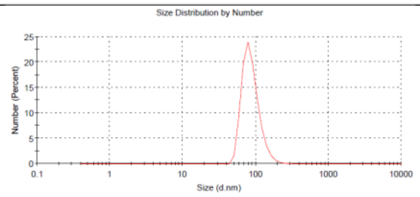

48 h / day 2

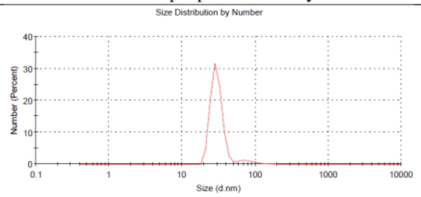

72 h / day 3

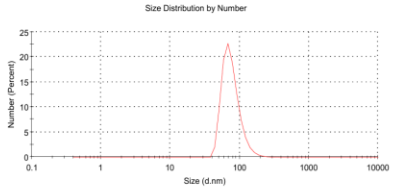

After one week

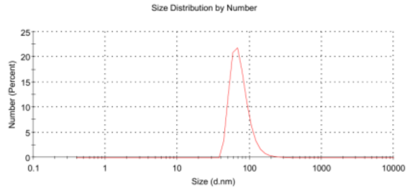

After two weeks

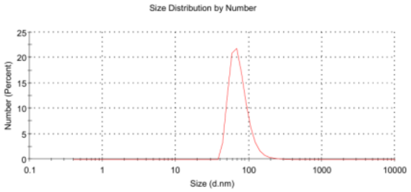

After three weeks

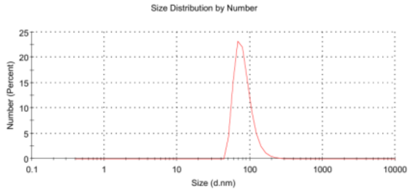

After four weeks

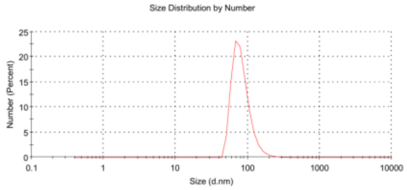

After five weeks

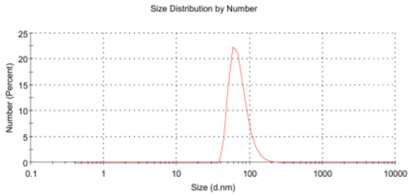

After six weeks

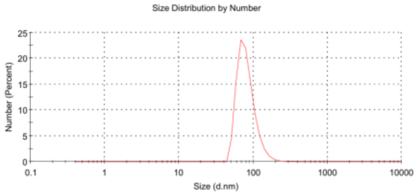

After seven weeks

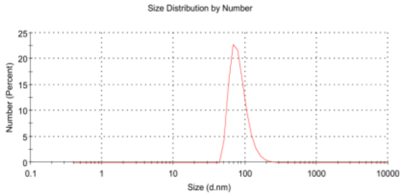

After eight weeks

Table S5. DLS spectra and size distributions by number recorded over 8 weeks for **LuPcPCL$_{50}$/10$_{from}$20**

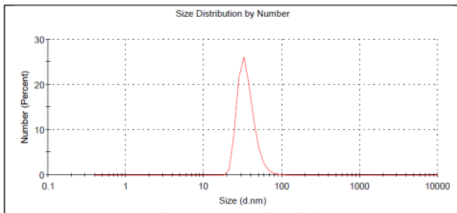

Just after preparation / day 0

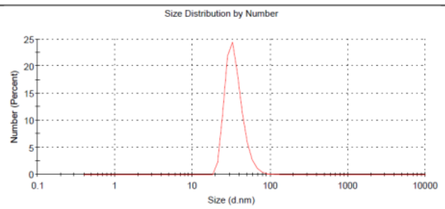

24 h / day 1

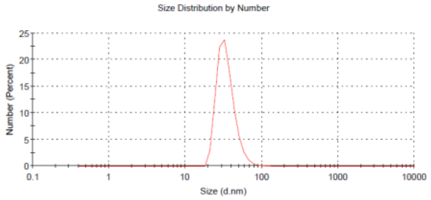

48 h / day 2

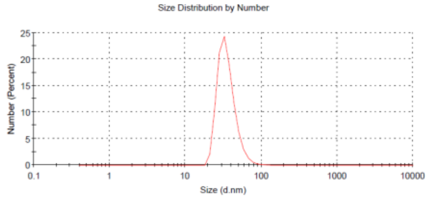

72 h / day 3

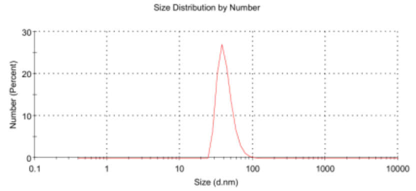

After one week

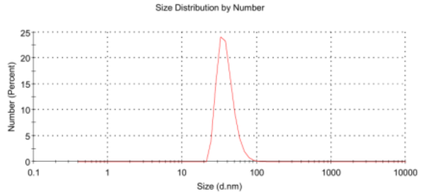

After two weeks

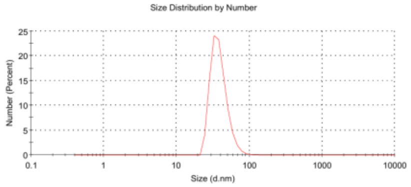

After three weeks

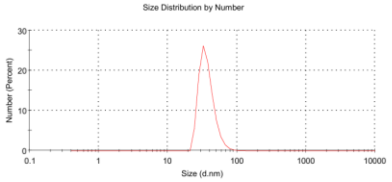

After four weeks

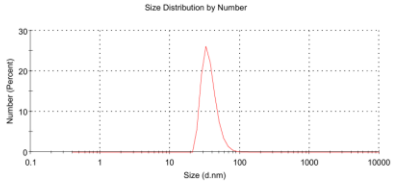

After five weeks

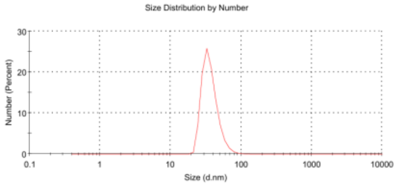

After six weeks

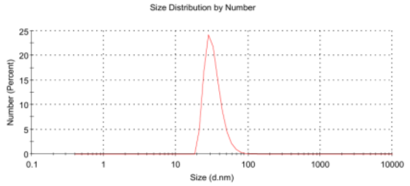

After seven weeks

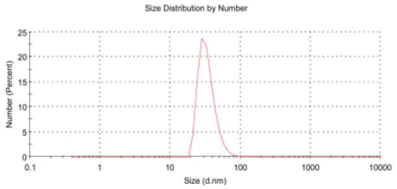

After eight weeks

Table S6. DLS spectra and size distributions by number recorded over 8 weeks for **LuPcPCL$_{50}$/10$_{from}$100**

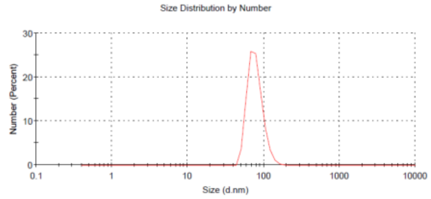

Just after preparation / day 0

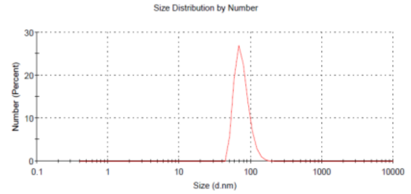

24 h / day 1

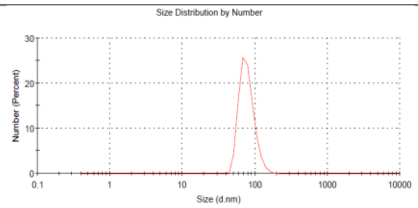

48 h / day 2

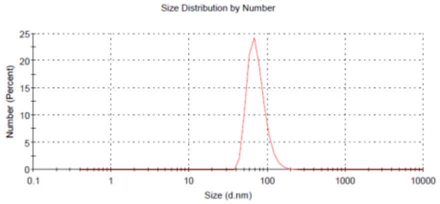

72 h / day 3

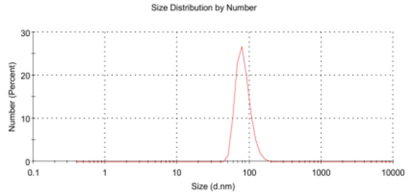

After one week

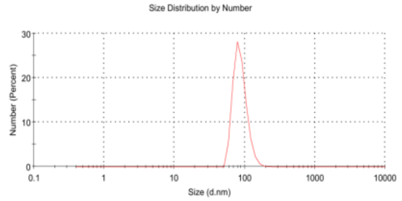

After two weeks

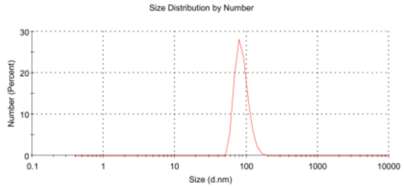

After three weeks

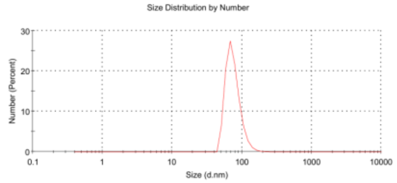

After four weeks

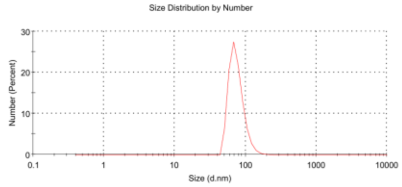

After five weeks

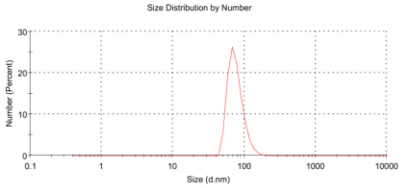

After six weeks

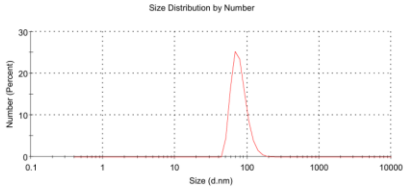

After seven weeks

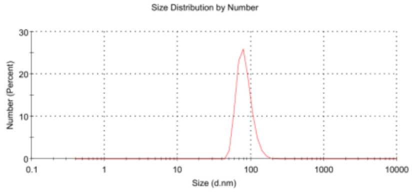

After eight weeks

Table S7. DLS spectra and size distributions by number recorded over 8 weeks for **LuPcPCL$_{50}$/50$_{from}$100**

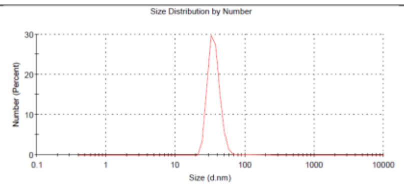

Just after preparation / day 0

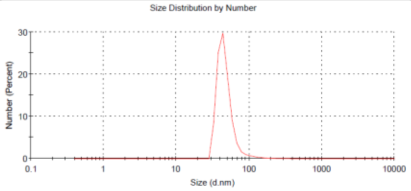

24 h / day 1

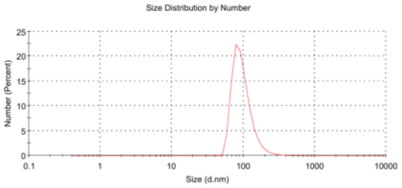

48 h / day 2

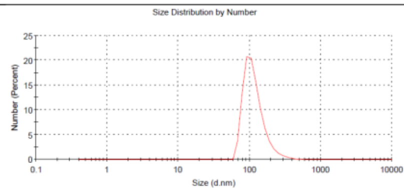

72 h / day 3

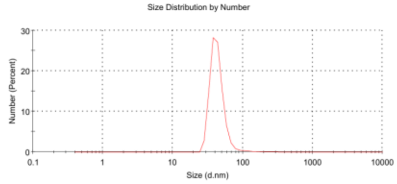

After one week

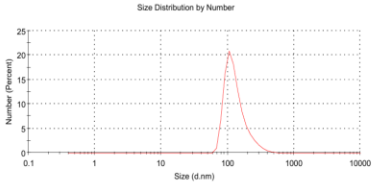

After two weeks

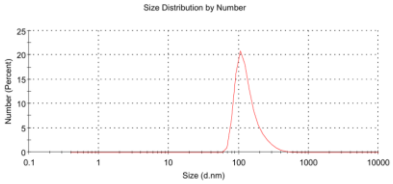

After three weeks

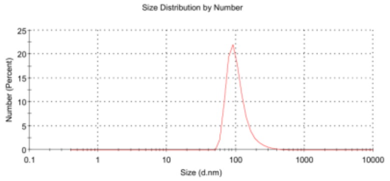

After four weeks

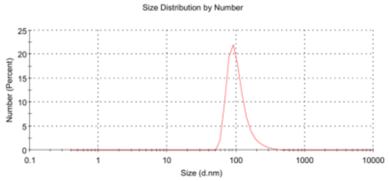

After five weeks

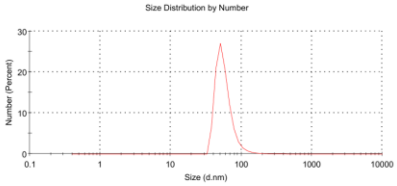

After six weeks

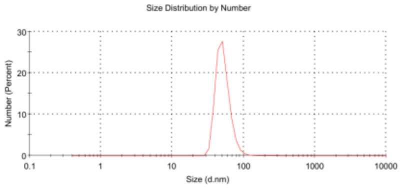

After seven weeks

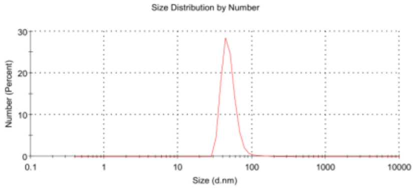

After eight weeks

Table S8. Zeta potential distribution spectra of the nanoparticles

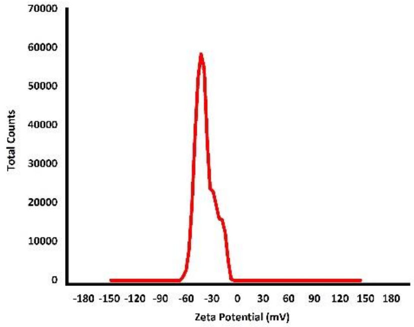

LuPcPCL$_{20}$/ 10$_{from}$20

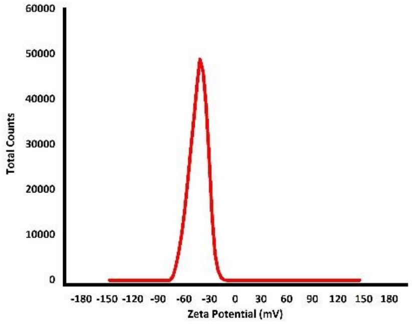

LuPcPCL$_{20}$/ 10$_{from}$100

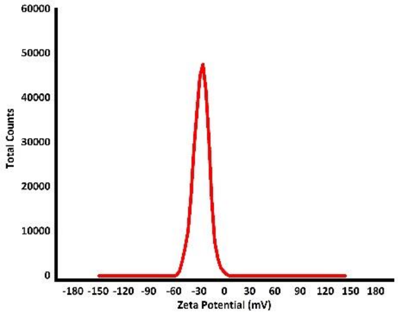

LuPcPCL$_{20}$/ 50$_{from}$100

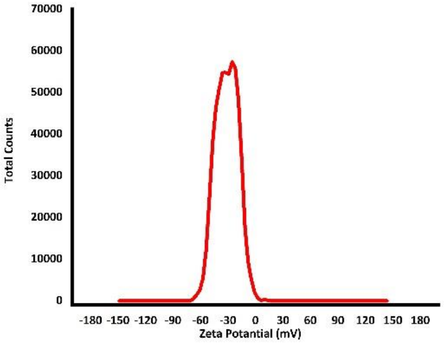

LuPcPCL$_{50}$/ 10$_{from}$20

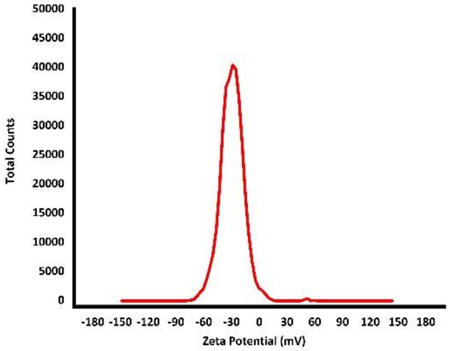

LuPcPCL$_{50}$/ 10$_{from}$100

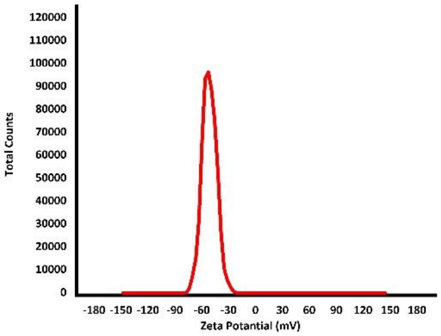

LuPcPCL50/ 50$_{from}$100
